# CEBPA基因突变在急性髓系白血病中的预后意义

**DOI:** 10.3760/cma.j.cn121090-20251111-00520

**Published:** 2026-05

**Authors:** 真 申, 风红 张, 文治 蔡, 萌 唐, 宏杰 沈, 小飞 杨, 苏宁 陈, 尚龙 冯

**Affiliations:** 苏州大学附属第一医院血液内科，江苏省血液研究所，国家血液系统疾病临床医学研究中心，苏州 215006 Department of Hematology, the First Affiliated Hospital of Soochow University, Jiangsu Institute of Hematology, National Clinical Research Center for Hematologic Diseases, Suzhou 215006, China

**Keywords:** 白血病，髓样，急性, CEBPA基因突变, 化疗, 预后, Leukemia, myeloid, acute, CEBPA gene mutation, Chemotherapy, Prognosis

## Abstract

**目的:**

探讨CEBPA基因突变在初诊急性髓系白血病（AML）患者中的预后价值。

**方法:**

本回顾性队列研究分析2016年1月至2024年12月苏州大学附属第一医院收治的237例接受强化诱导化疗的CEBPA突变阳性AML患者的临床资料，对比不同突变亚组的临床特征、治疗反应及预后。

**结果:**

237例患者中，bZIP结构域插入缺失突变（bZIP^InDel^）组182例（76.8％），bZIP结构域错义突变（bZIP^ms^）组13例（5.5％），其他类型突变（Other^mut^）组42例（17.7％）。与后两组相比，bZIP^InDel^组患者发病年龄更低（*P*<0.001），GATA2突变率更高（*P*<0.001），NPM1与DNMT3A突变率更低（*P*值均<0.05）。2个疗程诱导治疗后，bZIP^InDel^组完全缓解（CR）/CR伴血细胞不完全恢复（CRi）率及可检测残留病（MRD）阴性率均显著高于bZIP^ms^组和Other^mut^组（CR/CRi率：99.4％对75.0％对86.8％，*P*<0.001；MRD阴性率：88.6％对77.8％对66.7％，*P*＝0.008）。中位随访41个月，bZIP^InDel^组3年总生存（OS）率及无复发生存（RFS）率分别为90.7％及72.1％。多因素分析显示，CEBPA bZIP^InDel^是OS（*HR*＝0.16，95％*CI*：0.06～0.40，*P*<0.001）与RFS（*HR*＝0.31，95％*CI*：0.17～0.57，*P*<0.001）的独立良好预后因素。bZIP^InDel^组中，1个疗程达CR/CRi者的3年OS率优于2个疗程达CR/CRi者（88.3％对48.9％，*P*＝0.050）；首次CR（CR1）期行异基因造血干细胞移植虽可显著改善3年RFS率（89.7％对57.6％，*P*<0.001），但未能转化为OS获益（*P*＝0.376）。此外，合并KIT突变与较低的3年OS率相关（65.6％对91.7％，*P*＝0.042），合并CSF3R突变与较低的3年RFS率相关（42.9％对75.1％，*P*<0.001）。

**结论:**

CEBPA bZIP^InDel^具有独特的预后价值，该亚组化疗获益显著，但伴随KIT或CSF3R突变者常预后不佳。

## Abstract (en)

**Objective:**

To investigate the prognostic value of CEBPA gene mutations in patients with newly diagnosed acute myeloid leukemia（AML）.

**Methods:**

A retrospective cohort analysis was conducted on the clinical data from 237 CEBPA-mutated AML patients who received intensive chemotherapy induction at the First Affiliated Hospital of Soochow University between January 2016 and December 2024. Clinical characteristics, treatment responses, and prognosis were compared among different mutation subgroups.

**Results:**

Among 237 patients, 182（76.8％）harbored bZIP domain in-frame insertion/deletion mutations（bZIP^InDel^）, 13（5.5％）had bZIP domain missense mutations（bZIP^ms^）, and 42（17.7％）carried other mutation types（Other^mut^）. Compared with the latter two groups, patients in the bZIP^InDel^ group had a younger age at onset（*P*<0.001）, a higher GATA2 mutation rate（*P*<0.001）, and lower mutation rates of NPM1 and DNMT3A（both *P*<0.05）. After two courses of induction therapy, the bZIP^InDel^ group achieved significantly higher complete remission（CR）/CR with incomplete hematologic recovery（CRi）rates and measurable residual disease（MRD）-negative rates than the bZIP^ms^ and Other^mut^ groups（CR/CRi rates: 99.4％ *vs* 75.0％ *vs* 86.8％, *P*<0.001; MRD-negative rates: 88.6％ *vs* 77.8％ *vs* 66.7％, *P*＝0.008）. With a median follow-up of 41 months, the 3-year overall survival（OS）and relapse-free survival（RFS）rates in the bZIP^InDel^ group were 90.7％ and 72.1％, respectively. Multivariate analysis revealed that CEBPA bZIP^InDel^ was an independent favorable prognostic factor for both OS（*HR*＝0.16, 95％*CI*: 0.06–0.40, *P*<0.001）and RFS（*HR*＝0.31, 95％*CI*: 0.17–0.57, *P*<0.001）. Within the bZIP^InDel^ group, patients achieving CR/CRi after one course of induction had a superior 3-year OS rate compared with those achieving CR/CRi after two courses（88.3％ *vs* 48.9％, *P*＝0.050）. Although allogeneic hematopoietic stem cell transplantation performed during the first CR（CR1）significantly improved the 3-year RFS rate（89.7％ *vs* 57.6％, *P*<0.001）, this benefit did not translate into an OS advantage（*P*＝0.376）. Additionally, concurrent KIT mutations were associated with a lower 3-year OS rate（65.6％ *vs* 91.7％, *P*＝0.042）, and concurrent CSF3R mutations were associated with a lower 3-year RFS rate（42.9％ *vs* 75.1％, *P*<0.001）.

**Conclusion:**

CEBPA bZIP^InDel^ confer a distinct prognostic value. Patients in this subgroup derive substantial benefit from chemotherapy; however, the presence of concurrent KIT or CSF3R mutations is frequently associated with unfavorable outcomes.

CEBPA基因所编码的蛋白由转录激活结构域（TAD）和碱性亮氨酸拉链（bZIP）结构域组成，是调控髓系分化的关键转录因子[1]。该基因突变在急性髓系白血病（AML）中的发生率为5％～10％，其预后意义在欧洲白血病网（ELN）指南的历次更新中逐步精确：2010版将“伴正常核型的CEBPA突变”列为预后良好因素，2017版明确限定为“CEBPA双突变”，2022版进一步聚焦于“bZIP区框内突变（包括插入/缺失与错义突变）”[2]–[6]。国外近期研究发现，CEBPA突变带来的良好预后可能仅与bZIP区的插入/缺失突变（InDel）相关[7]。为在中国AML患者中验证上述结论并探讨其临床价值，本研究回顾性纳入2016年1月至2024年12月于苏州大学附属第一医院血液内科接受强化疗的237例CEBPA突变AML患者，旨在系统评估CEBPA不同突变类型、相应治疗策略及合并突变对预后的影响。

## 病例与方法

1. 临床资料：本回顾性队列研究纳入2016年1月至2024年12月在苏州大学附属第一医院接受强化诱导化疗的237例CEBPA基因突变阳性AML（非急性早幼粒细胞白血病）患者。本研究经苏州大学附属第一医院伦理委员会批准（批件号：20251045）。所有入组患者均完成骨髓细胞形态学、免疫分型、染色体核型分析、融合基因及基因突变检测。患者诊断均依据2016版世界卫生组织白血病分类标准[8]，治疗方案参考《成人急性髓系白血病（非急性早幼粒细胞白血病）中国诊疗指南（2011年版）》[9]。所有患者均接受强化诱导化疗，主要方案包括：IA方案（去甲氧柔红霉素8～12 mg/m^2^，第1～3天；阿糖胞苷100～200 mg/m^2^，第1～7天）、DA方案（柔红霉素60～90 mg/m^2^，第1～3天；阿糖胞苷100～200 mg/m^2^，第1～7天）及IAC方案（去甲氧柔红霉素8 mg/m^2^，第1～3天；阿糖胞苷100 mg/m^2^，第1～7天；克拉屈滨5 mg/m^2^，第1～5天）。巩固治疗方案包括以中、大剂量阿糖胞苷（1～3 g/m^2^，每12 h 1次，第1～3天）为基础的化疗或造血干细胞移植。

2. 二代测序：采集初诊患者治疗前骨髓液3 ml，分离单个核细胞后，使用Invitrogen DNA提取试剂盒提取基因组DNA，并通过Ion GeneStudio S5系统进行测序。本研究共检测52种AML常见突变基因，包括：ASXL1、ASXL2、BCOR、BCORL1、BIRC3、BRAF、CALR、CBL、CDKN2A、CEBPA、CSF3R、CSMD1、DNMT3A、ETNK1、ETV6、EZH2、FBXW7、FLT3、GATA2、IDH1、IDH2、IL7R、JAK1、JAK2、JAK3、KIT、KRAS、MPL、MYD88、NF1、NOTCH1、NPM1、NRAS、PAX5、PDGFRA、PDGFRB、PHF6、PIGA、PTEN、PTPN11、RUNX1、SETBP1、SETD2、SF3B1、SH2B3、SRSF2、STAG2、TET2、TP53、U2AF1、WT1、ZRSR2。利用IGV软件进行测序结果可视化分析，并综合使用dbSNP、1000 Genomes、COSMIC、HGMD及本实验室自建数据库对变异进行注释。

3. 疗效评估：完全缓解（CR）定义为：骨髓原始细胞比例<5％，外周血中无原始细胞，无髓外病变，ANC≥1.0×10^9^/L，PLT≥100×10^9^/L。CR伴血细胞不完全恢复（CRi）定义为除ANC<1.0×10^9^/L或PLT<100×10^9^/L外，满足其余CR标准。可检测残留病（MRD）阴性定义为流式细胞术检测中具有目标免疫表型的CD45阳性细胞比例<0.1％。本研究的疗效评价（CR、MRD阴性）均以患者完成2个疗程诱导化疗后的评估结果为准。

4. 随访：通过查阅门诊与住院病历及电话随访收集患者的随访资料，随访截止日期为2025年7月31日，中位随访41（0～113）个月。总生存（OS）期指患者自确诊至死亡或末次随访的时间，无复发生存（RFS）期指自患者达到CR/CRi至复发、死亡或末次随访的时间。

5. 统计学处理：采用R语言（版本4.4.2）进行统计分析。定性资料用例数（百分比）表示，定量资料用*M*（范围）或*M*（*Q*_1_，*Q*_3_）表示。连续变量的组间比较采用*t*检验或Mann-Whitney *U*检验，分类变量的比较采用*χ*^2^检验或Fisher精确检验。采用Kaplan-Meier法绘制生存曲线，组间比较采用Log-rank检验。预后相关因素首先通过单因素Cox回归模型筛选，将*P*<0.1的变量纳入多因素Cox回归模型进行独立预后因素分析。所有统计检验均为双侧，*P*<0.05为差异有统计学意义。

## 结果

1. 基线特征：如表1所示，237例携带CEBPA突变的AML患者中，bZIP结构域插入缺失突变（bZIP^InDel^，包含单、双突变）最为常见［182例（76.8％）］，其次为其他类型突变（Other^mut^，包括bZIP区移码/无义突变及TAD区突变）［42例（17.7％）］，而bZIP结构域错义突变（bZIP^ms^）最少见［13例（5.5％）］。与bZIP^ms^及Other^mut^组相比，bZIP^InDel^组患者的初诊年龄更小（*P*<0.001），HGB水平更高（*P*＝0.013），PLT更低（*P*＝0.001），GATA2突变比例更高（*P*<0.001），NPM1（*P*<0.001）与DNMT3A（*P*＝0.004）突变比例更低。细胞遗传学分析显示，bZIP^InDel^组中132例（76.3％）患者为正常核型。根据ELN 2022风险分层标准（未纳入CEBPA突变状态），bZIP^InDel^组有143例（82.7％）患者被归为中危组。

**表1:** 不同CEBPA突变类型的急性髓系白血病患者临床特征比较

临床特征	bZIP^InDel^组（182例）	bZIP^ms^组（13例）	Other^mut^组（42例）	统计量	*P*值
年龄［岁，*M*（*Q*_1_, *Q*_3_）］	33（28，45）	39（32，47）	47（37，55）	26.700	<0.001
男性［例（％）］	104（57.1）	6（46.2）	22（52.4）	0.821	0.663
白细胞计数（×10^9^/L，*M*（*Q*_1_, *Q*_3_）］	16.7（8.1，66.9）	16.0（2.9，26.2）	17.5（3.2，89.2）	1.910	0.385
血红蛋白［g/L，*M*（*Q*_1_, *Q*_3_）］	103.0（85.5，117.2）	94.0（81.0，115.0）	89.0（72.5，103.2）	8.538	0.013
血小板计数［×10^9^/L，*M*（*Q*_1_, *Q*_3_）］	25.5（14.2，45.0）	35.0（22.0，115.0）	44.5（25.0，83.8）	15.000	0.001
骨髓原始细胞比例（％）	59.2（44.0，72.2）	54.3（41.0，62.0）	50.8（31.0，70.8）	3.960	0.138
核型［例（％）］				–	0.626
正常核型	132（76.3）	10（76.9）	27（69.2）		
其他核型	41（23.7）	3（23.1）	12（30.8）		
ELN 2022预后分层［例（％）］^a^				–	<0.001
低危组	0（0）	1（7.7）	8（20.5）		
中危组	143（82.7）	8（61.5）	25（64.1）		
高危组	30（17.3）	4（30.8）	6（15.4）		
合并基因突变［例（％）］				–	
GATA2	56（30.8）	1（7.7）	1（2.4）		<0.001
NPM1	0（0）	1（7.7）	13（31.0）		<0.001
DNMT3A	8（4.4）	2（15.4）	8（19.0）		0.004
FLT3-ITD	17（9.3）	3（23.1）	8（19.0）		0.051
TET2	15（8.2）	1（7.7）	8（19.0）		0.106
WT1	53（29.1）	4（30.8）	6（14.3）		0.123
NRAS/KRAS	31（17.0）	0（0）	7（16.7）		0.321
CSF3R	16（8.8）	1（7.7）	2（4.8）		0.740
KIT	9（5.0）	1（7.7）	4（9.5）		0.343
MR^b^	34（18.7）	4（30.8）	7（16.7）		0.523
CR/CRi［例（％）］				–	<0.001
是	177（99.4）	9（75.0）	33（86.8）		
否	1（0.6）	3（25.0）	5（13.2）		
MRD状态［例（％）］				–	0.008
阴性	148（88.6）	7（77.8）	20（66.7）		
阳性	19（11.4）	2（22.2）	10（33.3）		
首次CR期移植［例（％）］				1.230	0.542
是	80（44.0）	5（38.5）	22（52.4）		
否	102（56.0）	8（61.5）	20（47.6）		

**注** bZIP^InDel^：bZIP结构域插入缺失突变；bZIP^ms^：bZIP结构域错义突变；Other^mut^：其他类型突变，包括bZIP区移码/无义突变及TAD区突变；ELN：欧洲白血病网；CR：完全缓解；CRi：CR伴血细胞不完全恢复；MRD：可检测残留病；–：部分亚组例数较少，行Fisher精确检验，无统计量；^a^预后分层依据ELN 2022标准，其中未纳入CEBPA基因突变；^b^MR突变指骨髓增生异常相关突变，定义为ASXL1、BCOR、EZH2、RUNX1、SF3B1、SRSF2、STAG2、U2AF1、ZRSR2基因突变

2. CEBPA突变类型对预后的影响：经2个疗程诱导治疗后，bZIP^InDel^组患者的CR/CRi率（99.4％对75.0％对86.8％，*P*<0.001）及MRD阴性率（88.6％对77.8％对66.7％，*P*＝0.008）均显著高于bZIP^ms^组和Other^mut^组（表1）。

在长期预后方面，bZIP^InDel^与bZIP^ms^组均显示出生存优势（图1）。bZIP^InDel^与bZIP^ms^组患者的3年OS率（90.7％对100.0％对62.2％，*P*<0.001）及3年RFS率（72.1％对77.8％对59.5％，*P*＝0.063）均优于Other^mut^组。为排除异基因造血干细胞移植（allo-HSCT）的混杂影响，对首次CR（CR1）期接受移植的患者进行了删失处理，结果显示，bZIP^InDel^与bZIP^ms^组的2年OS率（92.1％对100.0％对49.7％，*P*<0.001）与2年RFS率（64.1％对68.6％对36.7％，*P*＝0.001）高于Other^mut^组（图2）。

**图1:**
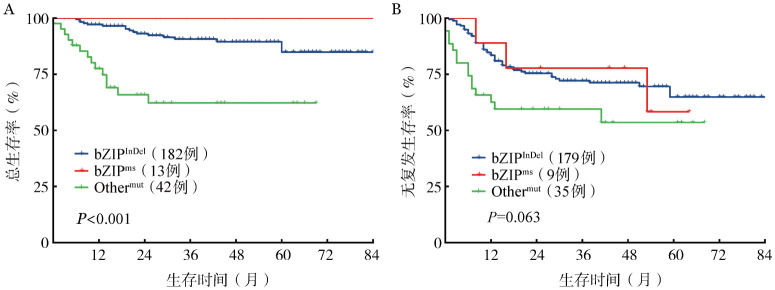
bZIP^InDel^、bZIP^ms^、Other^mut^组急性髓系白血病患者的总生存（A）和无复发生存（B）曲线 **注** bZIP^InDel^：bZIP结构域插入缺失突变；bZIP^ms^：bZIP结构域错义突变；Other^mut^：其他类型突变

**图2:**
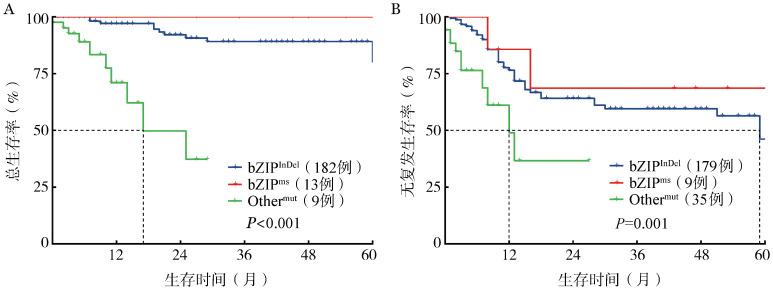
首次完全缓解期移植患者删失后bZIP^InDel^、bZIP^ms^、Other^mut^组急性髓系白血病患者的总生存（A）和无复发生存（B）曲线 **注** bZIP^InDel^：bZIP结构域插入缺失突变；bZIP^ms^：bZIP结构域错义突变；Other^mut^：其他类型突变

进一步通过Cox比例风险回归分析确定bZIP^InDel^与bZIP^ms^突变是否为患者的独立预后因素，纳入的协变量包括年龄、WBC、骨髓原始细胞比例、ELN 2022风险分层（剔除CEBPA突变状态）、CEBPA突变类型、WT1突变、NRAS/KRAS突变、诱导治疗后CR/CRi率、诱导治疗后MRD阴性率及CR1期接受移植，单因素分析结果见附表1（**扫描本文首页二维码查阅**）。多因素分析结果显示，CEBPA bZIP^InDel^（*HR*＝0.16，95％*CI*：0.06～0.40，*P*<0.001）、诱导治疗后达到CR/CRi（*HR*＝0.18，95％*CI*：0.05～0.70，*P*＝0.014）及CR1期接受移植（*HR*＝0.28，95％*CI*：0.12～0.65，*P*＝0.003）是OS的良好独立预后因素（表2）。CEBPA bZIP^InDel^（*HR*＝0.31，95％*CI*：0.17～0.57，*P*<0.001）与CR1期移植（*HR*＝0.13，95％*CI*：0.07～0.25，*P*＝0.001）同样为RFS的保护因素；较高的WBC（*HR*＝1.01，95％*CI*：1.00～1.01，*P*<0.001）是RFS的独立不良预后因素（表2）。

**表2:** 影响CEBPA突变的急性髓系白血病患者总生存和无复发生存的多因素分析

变量	总生存			无复发生存		
	*HR*	95％*CI*	*P*值	*HR*	95％*CI*	*P*值
年龄（岁）	0.99	0.96～1.02	0.521	–	–	–
白细胞计数（×10^9^/L）	–	–	–	1.01	1.00～1.01	<0.001
骨髓原始细胞比例（％）	–	–	–	–	–	–
ELN 2022预后分层						
低危组对中危组	–	–	–	–	–	–
高危组对中危组	–	–	–	–	–	–
CEBPA突变类型						
bZIP^InDel^对Other^mut^	0.16	0.06～0.40	<0.001	0.31	0.17～0.57	0.001
bZIP^ms^对Other^mut^	–	–	–	0.31	0.09～1.10	0.071
WT1基因突变	–	–	–	–	–	–
GATA2基因突变	–	–	–	–	–	–
NRAS/KRAS基因突变	–	–	–	–	–	–
诱导治疗后CR/CRi	0.18	0.05～0.70	0.014	–	–	–
诱导治疗后MRD阴性	–	–	–	–	–	–
首次CR期移植	0.28	0.12～0.65	0.003	0.13	0.07～0.25	<0.001

**注** ELN：欧洲白血病网；bZIP^InDel^：bZIP结构域插入缺失突变；Other^mut^：其他类型突变，包括bZIP结构域移码/无义突变及TAD区突变；bZIP^ms^：bZIP结构域错义突变；CR：完全缓解；CRi：CR伴血细胞不完全恢复；MRD：可检测残留病；–：单因素分析中*P*≥0.1，未进行多因素分析

4. 预后的影响因素：bZIP^InDel^组患者接受1个疗程诱导化疗后CR/CRi率为89.9％（160/178）；2个疗程后，CR/CRi率提升至99.4％（177/178），其中88.6％（148/167）的患者实现了MRD阴性。在巩固治疗阶段，44.0％（78/177）的患者在CR1期接受了allo-HSCT。在CR1期移植删失患者中，1个疗程达CR/CRi者的3年OS率（88.3％对48.9％，*P*＝0.050）与3年RFS率（60.3％对24.1％，*P*＝0.020）优于2个疗程达CR/CRi者（图3）；然而，2个疗程后MRD阴性组与阳性组3年OS率（89.5％对90.0％，*P*＝0.940）和3年RFS率（61.9％对62.2％，*P*＝0.670）的差异均无统计学意义（图3）。对于患者总体，CR1期allo-HSCT显著改善了3年RFS率（89.7％对57.6％，*P*<0.001），但对3年OS率无显著影响（92.7％对88.9％，*P*＝0.376）（图3）。

**图3:**
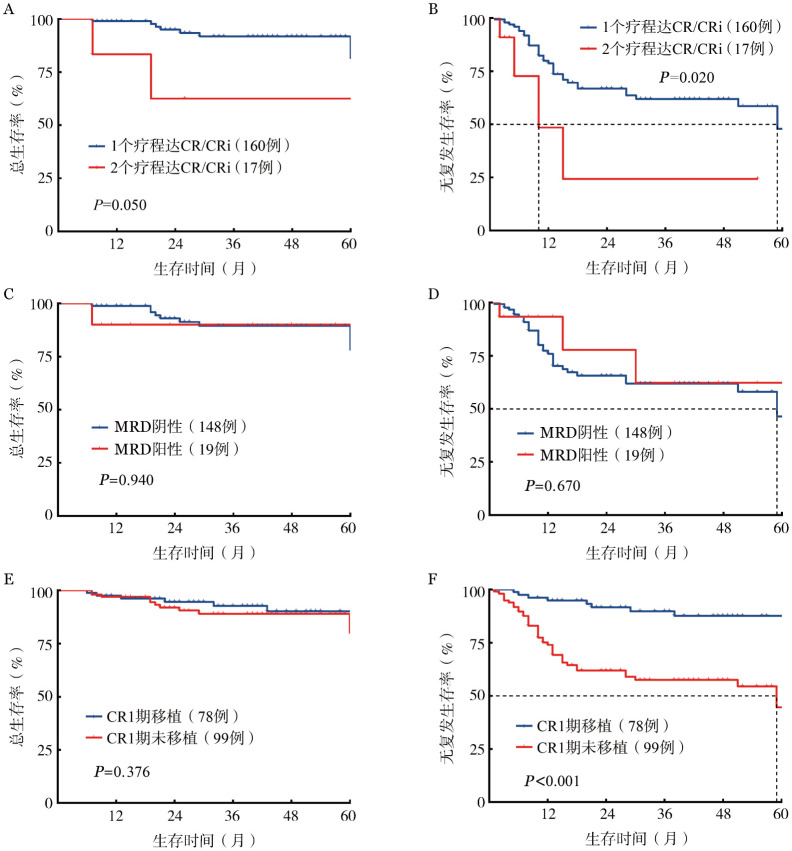
CEBPA bZIP结构域插入缺失突变急性髓系白血病患者的生存曲线 **A、B** 1个、2个疗程诱导化疗后达完全缓解（CR）/CR伴血细胞不完全恢复（CRi）患者的总生存和无复发生存曲线；**C、D** 可检测残留病（MRD）阴性、阳性患者的总生存和无复发生存曲线；**E、F** 首次CR（CR1）期移植与未移植患者的总生存和无复发生存曲线

根据ELN 2022指南，CEBPA bZIP区框内突变（包括bZIP^InDel^和bZIP^ms^）被归为低危组。本研究Cox多因素分析结果显示，仅bZIP^InDel^是OS和RFS的独立良好预后因素，而bZIP^ms^不影响预后。将bZIP^ms^组与Other^mut^组合并为Others组。Kaplan-Meier生存分析显示，在Others组中，2个疗程诱导治疗后达到CR/CRi（3年OS率：76.7％对60.0％，*P*＝0.039）及在CR1期接受allo-HSCT（3年OS率：84.7％对54.3％，*P*＝0.027）均与更好的OS相关（图4）。在CR1期接受allo-HSCT也显著改善了RFS（3年RFS率：87.0％对34.9％，*P*＝0.002）（图4）。

**图4:**
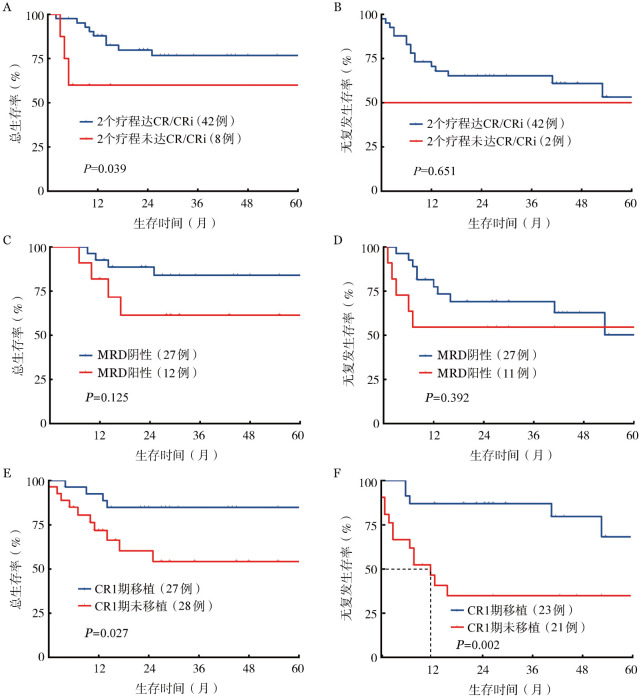
bZIP^ms^和Other^mut^组急性髓系白血病患者的生存曲线 **A、B** 是否达到完全缓解（CR）/CR伴血细胞不完全恢复（CRi）患者的总生存和无复发生存曲线；**C、D** 可检测残留病（MRD）阴性、阳性患者的总生存和无复发生存曲线；**E、F** 首次完全缓解（CR1）期移植与未移植患者的总生存和无复发生存曲线 **注** bZIP^ms^：bZIP结构域错义突变；Other^mut^：其他类型突变

Kaplan-Meier生存分析结果显示，合并KIT突变与较低的3年OS率相关（65.6％对91.7％，*P*＝0.042），合并CSF3R突变对3年RFS率有显著影响（42.9％对75.1％，*P*<0.001）。此外，合并WT1突变（3年OS率：86.6％对92.4％，*P*＝0.232；3年RFS率：70.7％对73.0％，*P*＝0.590）与骨髓增生异常相关基因突变（MR突变，包括SRSF2、SF3B1、U2AF1、ZRSR2、ASXL1、EZH2、BCOR、STAG2、RUNX1基因突变）（3年OS率：94.0％对89.8％，*P*＝0.410；3年RFS率：75.1％对71.5％，*P*＝0.629）均未显著影响OS或RFS（**扫描本文首页二维码查阅附图1、附图2**）。

## 讨论

本研究纳入了237例接受强化诱导化疗的CEBPA突变AML患者，疗效分析显示，bZIP^InDel^组患者展现出更好的治疗反应。Cox多因素回归分析进一步证实，bZIP^InDel^突变是OS与RFS的独立预后因素，与既往研究结果相符。Taube等[5]对240例CEBPA突变AML患者的分析表明，bZIP区框内突变（包括bZIP^InDel^与bZIP^ms^）是获得CR、改善OS和RFS的独立预后因素。Georgi等[7]的研究纳入1 010例患者，发现bZIP^InDel^（双、单突变）患者的中位OS期（214.9、126.4个月）和RFS期（152.0、63.9个月）均显著优于bZIP^ms^（双、单突变）患者（中位OS期：39.7、39.7个月，中位RFS期：15.0、16.3个月）（*P*值均<0.001），提示仅bZIP^InDel^突变具有明确的预后价值。从分子机制角度看，上述差异可能与两者在bZIP功能域中的分布特征有关：bZIP^ms^突变多集中于碱性区，其突变可能直接干扰DNA识别与结合过程；而bZIP^InDel^突变主要位于叉状区及亮氨酸拉链前段，可能通过调节蛋白质构象间接调节DNA结合特性或蛋白相互作用能力[10]–[11]。

本研究接受强化疗诱导治疗的182例bZIP^InDel^突变AML患者中，160例（89.9％）在1个疗程后达到CR/CRi，177例（99.4％）在2个疗程后达到CR/CRi，1个疗程达到CR/CRi患者的OS和RFS均显著优于2个疗程后达到CR/CRi的患者。在获得CR/CRi的患者中，148例（88.6％）在2个疗程后达到了MRD阴性，但MRD状态并未转化为生存获益，未显著改善OS或RFS，与Vonk等[12]的研究结论一致。该研究纳入144例来自HOVON（荷兰-比利时血液肿瘤协作组）和SAKK（瑞士临床癌症研究组）临床试验的CEBPA突变AML患者，其中68例患者具备完整的免疫表型资料，12例患者流式细胞术检测MRD阳性，但其累积复发率与OS率与MRD阴性组相比差异均无统计学意义[12]。同时，该研究也提示，CEBPA bZIP区突变的MRD状态与预后无明显相关性，说明该领域仍需进一步深入探索。尽管本研究78例（44.0％）的患者在CR1期接受了移植巩固治疗，且该措施可改善RFS，但OS未见显著获益。与此一致，Georgi等[7]的研究也显示，在CR1期进行移植未使bZIP^InDel^突变患者的OS获益（*HR*＝1.19，95％*CI*：0.81～1.75，*P*＝0.178），或许与该突变患者复发后再诱导治疗缓解率较高有关。值得注意的是，Lu等[13]的前瞻性随机临床试验显示，在CEBPA bZIP框内突变患者中，维奈克拉联合地西他滨诱导治疗的1年RFS率显著低于去甲氧柔红霉素联合阿糖胞苷方案（52.5％对85.1％，*P*＝0.017），并可能对OS产生不利影响（*HR*＝2.92）。因此，对于该基因亚型患者，在诱导与巩固治疗时均优先采用化疗策略。

本研究对CEBPA bZIP^InDel^突变组与其他CEBPA bZIP突变亚型的共存突变谱系进行分析，发现bZIP^InDel^突变主要与GATA2突变共存，而CEBPA其他突变亚型则更常伴随NPM1、DNMT3A和FLT3-ITD突变。目前，CEBPA合并突变对预后的影响尚存争议。在本研究的bZIP^InDel^队列中，Kaplan-Meier生存分析显示，合并KIT突变与患者OS期缩短显著相关，合并CSF3R突变显示出更差的RFS，这些发现表明，有必要探索联合靶向治疗方案改善该类患者的预后。而GATA2、WT1、TET2及MR突变则未对RFS或OS产生显著影响。值得注意的是，尽管有研究团队报道在CEBPA双突变患者中，GATA2突变与良好预后相关，FLT3-ITD和TET2突变提示预后不良，但该结论尚未在其他独立队列中获得一致性验证[5],[7],[14]–[18]。

综上，CEBPA bZIP^InDel^ AML具有独特的临床与生物学特征：患者初诊年龄更小、HGB水平更高、PLT更低；分子层面表现为GATA2共突变富集，而NPM1与DNMT3A共突变频率较低。该亚组患者对治疗反应良好，不仅CR/CRi率更高，OS期与RFS期也更具优势。多因素分析进一步证实，bZIP^InDel^是改善预后的独立影响因素。该亚组患者对化疗反应良好，缓解率显著，且展现出较优的长期OS与RFS；然而，合并KIT或CSF3R突变常预示预后不良，仍需优化治疗策略。本研究仍存在部分局限性，首先，本研究为单中心回顾性分析，仅纳入CEBPA突变阳性患者，缺乏与突变阴性患者的横向比较；bZIP^ms^组随访期间未观察到终点事件，导致Cox风险比例模型无法收敛，其预后优势可能未被充分量化评估；本研究病例数有限，样本量限制了部分亚组分析的统计效能。后续仍需通过多中心、前瞻性研究进一步验证相关结论，并深入探索其机制，以推动基于分子特征的精准预后分层与个体化治疗。

## Supplementary Material


