# Supplementary figures and images for: Prognostic significance of CEBPA gene mutations in acute myeloid leukemia
Source: Zhonghua Xue Ye Xue Za Zhi. 2026 May;47(5):450–7. [Article in Chinese] doi: 10.3760/cma.j.cn121090-20251111-00520 (PMC13416540; doi:10.3760/cma.j.cn121090-20251111-00520)

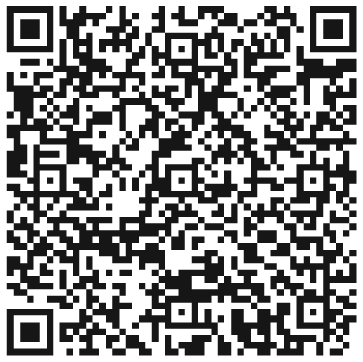

Supplement: Supplementary file 1 [file cjh-47-05-450-g005.tif]
